# 20α-Hydroxysteroid Dehydrogenase Expression in the Human Myometrium at Term and Preterm Birth: Relationships to Fetal Sex and Maternal Body Mass Index

**DOI:** 10.1007/s43032-023-01183-2

**Published:** 2023-02-10

**Authors:** Marina Paul, Tamas Zakar, Jason Phung, Amy Gregson, Anna Paredes Barreda, Trent A. Butler, Frederick R. Walker, Craig Pennell, Roger Smith, Jonathan W. Paul

**Affiliations:** 1 School of Biomedical Sciences and Pharmacy, College of Health, Medicine and Wellbeing, University of Newcastle, Callaghan, NSW 2308 Australia; 2 Hunter Medical Research Institute, New Lambton Heights, NSW 2305 Australia; 3 Centre for Rehab Innovations, University of Newcastle, Callaghan, NSW 2308 Australia; 4 School of Medicine and Public Health, College of Health, Medicine and Wellbeing, University of Newcastle, Callaghan, NSW 2308 Australia; 5 Mothers and Babies Research Centre, New Lambton Heights, NSW 2305 Australia; 6 John Hunter Hospital, New Lambton Heights, NSW 2305 Australia

**Keywords:** Myometrium, Progesterone, *AKR1C1*, 20α-HSD, Chorioamnionitis, BMI, Sex

## Abstract

The mechanism by which human labor is initiated in the presence of elevated circulating progesterone levels remains unknown. Recent evidence indicates that the progesterone-metabolizing enzyme, 20α-hydroxysteroid dehydrogenase (20α-HSD), encoded by the gene *AKR1C1*, may contribute to functional progesterone withdrawal. We found that *AKR1C1* expression significantly increased with labor onset in term myometrium, but not in preterm myometrium. Among preterm laboring deliveries, clinically diagnosed chorioamnionitis was associated with significantly elevated *AKR1C1* expression. *AKR1C1* expression positively correlated with BMI before labor and negatively correlated with BMI during labor. Analysis by fetal sex showed that *AKR1C1* expression was significantly higher in women who delivered male babies compared to women who delivered female babies at term, but not preterm. Further, in pregnancies where the fetus was female, *AKR1C1* expression positively correlated with the mother’s age and BMI at the time of delivery. In conclusion, the increase in myometrial *AKR1C1* expression with term labor is consistent with 20α-HSD playing a role in local progesterone metabolism to promote birth. Interestingly, this role appears to be specific to term pregnancies where the fetus is male. Upregulated *AKR1C1* expression in the myometrium at preterm in-labor with clinical chorioamnionitis suggests that increased 20α-HSD activity is a mechanism through which inflammation drives progesterone withdrawal in preterm labor. The link between *AKR1C1* expression and maternal BMI may provide insight into why maternal obesity is often associated with dysfunctional labor. Higher myometrial *AKR1C1* expression in male pregnancies may indicate fetal sex-related differences in the mechanisms that precipitate labor onset at term.

**Supplementary Information:**

The online version contains supplementary material available at 10.1007/s43032-023-01183-2.

## Introduction

Preterm birth is a major societal and economic problem that occurs in 5–18% of pregnancies and affects ~15 million pregnancies annually. Premature birth contributes to an estimated 1 million perinatal deaths per year [1], and this number is rising. The prevention of preterm birth is a major health priority [2]. Current therapy to block preterm labor fails to increase gestational length sufficiently to avoid prematurity and improve neonatal outcomes. The development of effective tocolysis is constrained by the shortage of knowledge about the mechanisms that drive term and preterm labor. There is ample evidence; however, highlighting the importance of progesterone (P4) in maintaining the pregnant state by promoting myometrial relaxation [3–5]. The withdrawal of P4 action signals the end of pregnancy, and in most mammalian species this occurs through a fall in circulating levels of P4, which precipitates labor [6–10]. In humans and higher primates, maternal, fetal, and amniotic concentrations of P4 remain elevated up to and during labor [11–13]. Nonetheless, blocking the actions of P4 by progesterone antagonist (e.g., RU486) promotes cervical ripening and labor in women [14, 15]. This indicates that P4 action is essential for maintaining human pregnancy and P4 withdrawal at parturition is functional occurring without a decline in the circulating hormone level.

Nadeem et al. [16] recently published new evidence of the molecular mechanisms of functional P4 withdrawal in the human myometrium. They found that P4 receptor (PR) complexes were undetectable in laboring myometrium by proximity ligation, while they were present in myometrial cell nuclei before labor at term [16]. Further, P4 abundance diminished in myometrial cell nuclei in labor but nuclear PR proteins remained detectable [16]. Follow-up experiments using cell culture models where PR-A and PR-B expressions could be selectively induced revealed that in the low P4 environment, unliganded PR-A was present in nuclei while PR-B partitioned in the cytoplasm [16]. Furthermore, reduced P4 abundance and appearance of unliganded PR-A in the nuclei were associated with the increased expression of the P4-metabolizing enzyme, 20α-hydroxysteroid dehydrogenase (20α-HSD), in the myometrial cells [16]. These observations agree with the reported pro-inflammatory actions of PR-A promoting labor and the central role of PR-B in maintaining P4-dependent myometrial quiescence [17]. They also suggest that the local metabolism of P4 by upregulated 20α-HSD contributes significantly to the withdrawal of P4 action in the laboring myometrium.

20α-HSD, a member of the aldo-keto reductase (AKR) superfamily, catalyzes the conversion of P4 to its inactive metabolite, 20α-hydroxyprogesterone (20α-OHP) [18]. The enzyme is encoded by the *AKR1C1* gene, which, in humans, is located on chromosome 10p15-p14. Piekorz et al. [19] demonstrated that mice lacking 20α-HSD have significantly delayed parturition despite a marked decline in circulating P4 levels. More recently, Williams et al. [20] showed that microRNA (miRNA)-200s directly target the transcription factor STAT5b, which is a negative regulator of 20α-HSD. They found that throughout most of the pregnancy, increased P4 levels inhibit miR-200 expression in the myometrium, which in turn allows upregulation of STAT5b and therefore inhibition of 20α-HSD expression [20, 21]. The miR-200 expression is upregulated in mouse myometrium near term and in human myometrium during labor, which leads to inhibition of STAT5b, thus permitting induction of 20α-HSD [20, 21]. 20α-HSD catalyzes the metabolism of P4, which decreases local P4 levels leaving PR-A unliganded to drive the expression of genes that promote labor [20, 21]. These findings highlight the involvement of 20α-HSD in P4 withdrawal by target tissue metabolism. 20α-HSD inhibition may be useful for maintaining P4 levels and prevent myometrial activation before term, but further studies are needed to explore this possibility. In the present study we determined *AKR1C1* gene expression in the human myometrium at term and preterm parturition and in relation to fetal sex, maternal body mass index (BMI), and maternal age.

## Materials and Methods

### Myometrial Tissue Acquisition

The study was approved by the Hunter and New England Area Human Research Ethics Committee (2019/ETH12330) and all participants gave informed written consent. Human myometrial samples were obtained from the upper lip of an incision in the lower uterine segment during the cesarean section of singleton preterm and term pregnancies that were either not-in-labor (NIL) or in-labor (IL) (Table 1). Women in labor were identified by the presence of regular, painful uterine contractions with evidence of cervical effacement and dilation over two vaginal examinations, or if the cervix was more than 4 cm dilated on one examination [22]. The diagnosis of clinical chorioamnionitis (preterm IL myometrium only) was made by clinical assessment, including fever >38°C/>100.4°F on two or more occasions, maternal tachycardia >100 beats per min (bpm), fetal tachycardia >160 bpm, uterine tenderness, purulent-appearing vaginal discharge, or elevated white cell count (>15,000/mm^2^). Patients with clinical chorioamnionitis also had histopathology demonstrating acute histologic chorioamnionitis. Maternal BMI data were collected during the first antenatal visit (second trimester, <20 weeks of fetal gestation). Common pregnancy complications were also recorded, such as intrauterine growth restriction (IUGR) and gestational diabetes mellitus (GDM). All myometrial samples were promptly washed in ice-cold phosphate-buffered saline (PBS), snap-frozen in liquid nitrogen and stored at −80°C. All myometrium samples were processed within 4 weeks of collection.

**Table 1:** Clinical characteristics of human myometrial samples

**Sample characteristics**	Preterm NIL	**Myometrial samples**			
		Preterm IL		Term NIL	Term IL
		No chorioamnionitis	Chorioamnionitis		
Number of patients	24	8	6	28	12
Gestational age (weeks)	27.0–36.5	27.0–36.3	27.3–31.4	38.0–41.0	37.5–41.6
BMI	16.7–48.3	20.8–43.4	20.8–34.2	18.3–38.0	19.1–36.4
Mother’s age (years)	17–37	20–34	25–42	19–40	20–36
Baby’s weight (g)	525–3,240	1,015–2,920	1,150 – 1,870	2,500 -4,350	2,640–4,970
Baby’s sex (male/female)	14/10	6/2	4/2	10/18	7/6
IUGR	Yes; *n*=15	No	No	No	No
Presence of GDM	Yes; *n*=3	No	Yes; *n*=1	No	Yes; *n*=1

### RNA Extraction, Reverse Transcription, and Real-time Quantitative PCR

RNA was extracted using TRizol Reagent (ThermoFisher) according to the manufacturer’s protocol. Homogenization of tissue in TRizol Reagent was performed using a Precellys24 homogenizer (5000 rpm for 3 × 30 sec, with 20 sec intervals) (Bertin Instruments). Following extraction, RNA samples were further purified using the TURBO DNA-*free* kit (ThermoFisher). RNA concentration (absorbance at 260 and 280 nm) and purity were assessed using a ND-1000 spectrophotometer and RNA integrity was checked by agarose gel electrophoresis before and after DNase treatment. Each RNA sample (0.5 μg of total RNA) was spiked with 0.5 × 10^7^ copies of Alien RNA (Integrated Sciences Pty) and reverse-transcribed using the SuperScript III First-Strand Synthesis System with random hexamer primers (ThermoFisher). Quantitative RT-PCR was performed using QuantStudio 6 Flex Real-Time PCR (Applied Biosystems). No-reverse transcription negative controls were prepared for each sample. The final volume of each PCR reaction was 20 μL, containing 10 μL of 2× SYBR Green PCR Master Mix (ThermoFisher), master mix cDNA template (corresponding to 10 ng of reverse-transcribed RNA), *AKR1C1* cDNA-specific forward and reverse primers (500 nM each), and MilliQ water to the final volume. For the reference cDNA (Alien), 1.0 μL of 2.5 μM of Alien Primer Mix (with proprietary sequence), 10 μL of 2× SYBR Green PCR Master Mix, and the same amount of cDNA as the target genes and MilliQ water were added up to the 20 μL final volume. No template samples were included in each PCR plate to detect any contamination and primer dimers. *AKR1C1* cDNA primers (Sigma) were designed using Primer-BLAST, optimized, and validated by confirming that single amplicons of appropriate size were generated (Table 2).

**Table 2:** cDNA primer sequence – *AKR1C1*

**Primer**	**Primer sequence**	**Amplicon size**	**GeneBank#**
*AKR1C1*	F: AGTTCACCGCTCGCATAA R: GGCTGTAGATAGGCTTAGTGT	60	NM_001353.6

### Protein Extraction, One-Dimensional (1D) SDS-PAGE, and Immunoblotting

Protein was extracted into sodium dodecyl sulfate (SDS) extraction buffer (2% SDS, 50 mM Tris pH 6.8, 5 mM EDTA) supplemented with PhosSTOP phosphatase inhibitor (Roche) and Complete Mini Protease Inhibitor (Roche). Tissue was homogenized in SDS extraction buffer using a Precellys24 homogenizer (6500 rpm for 3 × 60 sec, with 20 sec intervals), after which homogenates were incubated on a rotary mixer for 1 h at 4°C. Homogenates were then centrifuged at 15,500 g for 15 min at 4°C (Beckman Coulter Microfuge 20R) then supernatants collected. Protein concentration was determined using a BCA Protein Assay Kit (ThermoFisher Scientific).

Myometrial protein extracts (up to 50 μg per lane, due to low AKR1C1 abundance in pregnant human myometrium) were loaded onto 4–12% NuPAGE gels and separated using a Novex Mini-Cell system at constant voltage (200 V for 50 min; Invitrogen). S9 Fraction from Human Liver Extract (Sigma Aldrich, cat# S2442; up to 5.0 μg/lane) and SDS protein extract from term human placenta (up to 50 μg/lane) were included as positive and negative controls for AKR1C1 detection, respectively. Novex™ Sharp Pre-stained Protein Standard (10 μL/lane) was utilized as the molecular weight marker (ThermoFisher, cat# LC5800). Following 1D SDS-PAGE, proteins were transferred to Hybond-C nitrocellulose (Amersham Biosciences) using the XCell II Blot Module (Invitrogen). Total protein was stained using Ponceau S and imaged using an Amersham Imager 600 (GE Healthcare).

During immunoblotting, all incubations were performed on a rocking platform. Membranes were blocked in 5% skim milk powder in tris-buffered saline (TBS; 500 mM NaCl, 20 mM Tris) for 1 h at room temperature. Blocking solution was decanted and primary antibody applied in 5 mL 1% skim milk powder in TBS-Tween-20 (TBS-T; 500 mM NaCl, 20 mM Tris, 0.01% Tween-20) overnight at 4°C. Antibodies against AKR1C1 (cat# GTX105620, GeneTex, rabbit polyclonal; and cat# PA5-84776, ThermoFisher, rabbit polyclonal) and a β-actin (cat# ab8226, Abcam, mouse monoclonal) were applied at 1∶1000 dilution. Blots were subjected to 4 × 5 min washes with 100 mL of TBS-T. Washed blots were then incubated in horseradish peroxidase (HRP)-conjugated anti-rabbit IgG (cat# 7074, Cell Signaling) or anti-mouse IgG (cat# 7076, Cell Signaling) secondary antibody as appropriate, applied at 1∶2000 dilution in 5 mL of 1% skim milk powder in TBS-T for 1 h at room temperature. Blots were washed for 3 × 5 min in 100 mL of TBS-T before immunoreactive products were detected using Immobilon Forte (high sensitivity) Western HRP substrate (Millipore) and visualized using the Amersham Imager 600 (GE Healthcare). To ensure internal controls, blots were first probed with the AKR1C1 antibody before being stripped and re-probed with antibody against the total proteins. Membranes were stripped by 2×5 min incubations in 100 mL of 0.2 M NaOH. Stripped blots were then washed for 3×3 min in TBS-T before being re-probed according to the outlined regimen.

### Data and Statistical Analysis

All mRNA abundance data were expressed relative to the Alien reference RNA. The relative mRNA abundance was calculated using the delta Ct (∆Ct) method [23]. All mRNA relative abundance values were checked by the Shapiro-Wilk distribution test and not normally distributed data were logarithmically transformed to approach a normal distribution. Statistical analyses were conducted with GraphPad Prism software (California, USA) and confirmed using STATA (College Station, Texas, USA). Graphical data are presented as mean ± SEM. For comparison between two groups, Student’s *t*-test was used. For comparisons of multiple groups and interactions, analysis of variance and covariance (ANOVA) were performed. For mixed-effect analysis, two-way ANOVA (with repeated measures) followed by a post-hoc test of Sidak multiple comparisons were used. Correlations were analyzed by Pearson’s correlation. *P*-values <0.05 were considered statistically significant.

## Results

### *AKR1C1* mRNA Expression in Human Myometrium

**Fig. 1:**
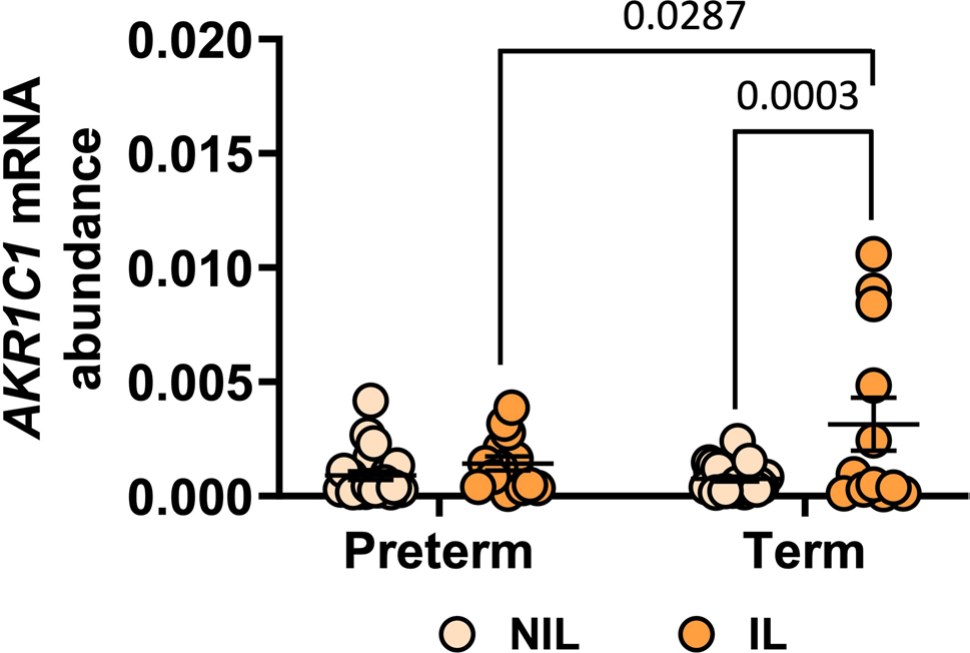
Expression of *AKR1C1* in preterm and term pregnant human myometrium. Relative abundance of *AKR1C1* mRNA was measured in myometrium biopsied from pregnant women who delivered preterm not-in-labor (NIL, *n*=24), preterm in-labor (IL, *n*=14), term not-in-labor (NIL, *n*=28), or term in-labor (IL, *n*=12). *AKR1C1* mRNA abundance is expressed relative to Alien reference. *Data were analyzed by two-way ANOVA with multiple comparisons (Sidak)*. Significant (*p*) values are indicated

*AKR1C1* expression was detected in both preterm and term myometrium before and during labor. Among the preterm samples, we detected no significant difference in *AKR1C1* mRNA abundance between preterm NIL (*n*=24) and preterm IL myometrium (*n*=14) (*p*=0.5976) (Fig. 1). Among the term samples, *AKR1C1* mRNA abundance was significantly higher in term IL myometrium (*n*=12) than in term NIL myometrium (*n*=28) (*p=*0.0003) (Fig. 1). Comparing across preterm and term samples, there was no significant difference in *AKR1C1* mRNA abundance between preterm NIL (*n*=24) and term NIL (*n*=28) myometrium (*p*=0.9442); however, *AKR1C1* mRNA abundance was significantly higher in term IL (*n*=12) compared to preterm IL myometrium (*n*=14) (*p=*0.0287) (Fig. 1).

**Fig. 2:**
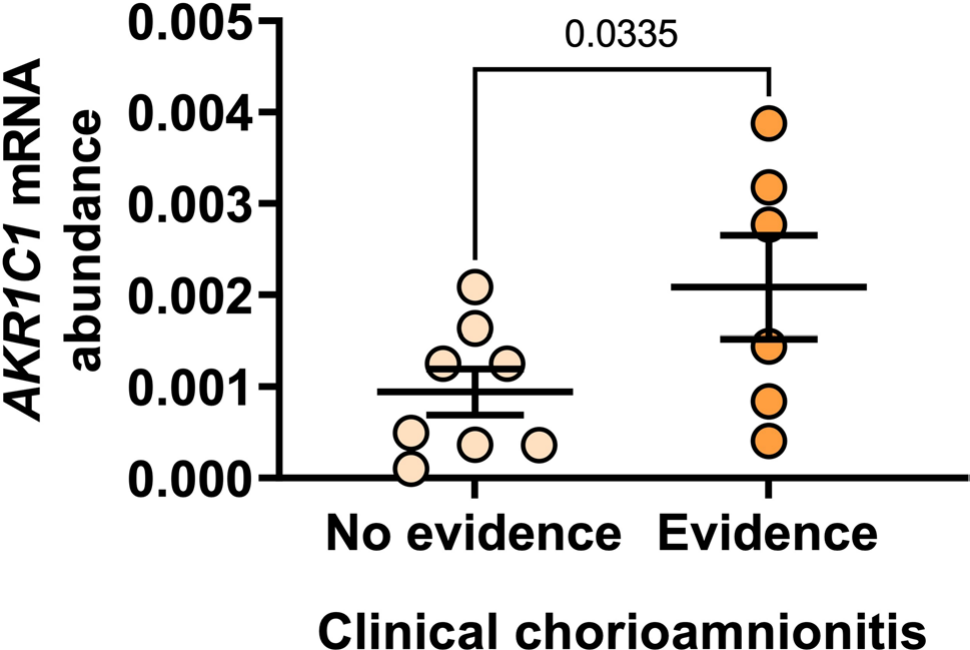
Myometrial *AKR1C1* expression from preterm IL deliveries without and with evidence of chorioamnionitis. Relative abundance of *AKR1C1* mRNA was measured in myometrium from preterm IL deliveries without (*n*=8) and with (*n*=6) evidence of clinical chorioamnionitis. *AKR1C1* mRNA abundance is expressed relative to Alien reference. *Data were analyzed by unpaired t-test*. Significant (*p*) values are indicated

Upon dividing preterm IL myometrium into those obtained from women with or without clinical evidence of chorioamnionitis, we detected significantly higher *AKR1C1* mRNA abundance in samples obtained from women diagnosed with clinical chorioamnionitis (*n*=6) compared to women with no evidence of intrauterine inflammation (*n*=8) (*p=*0.0335) (Fig. 2).

**Fig. 3:**
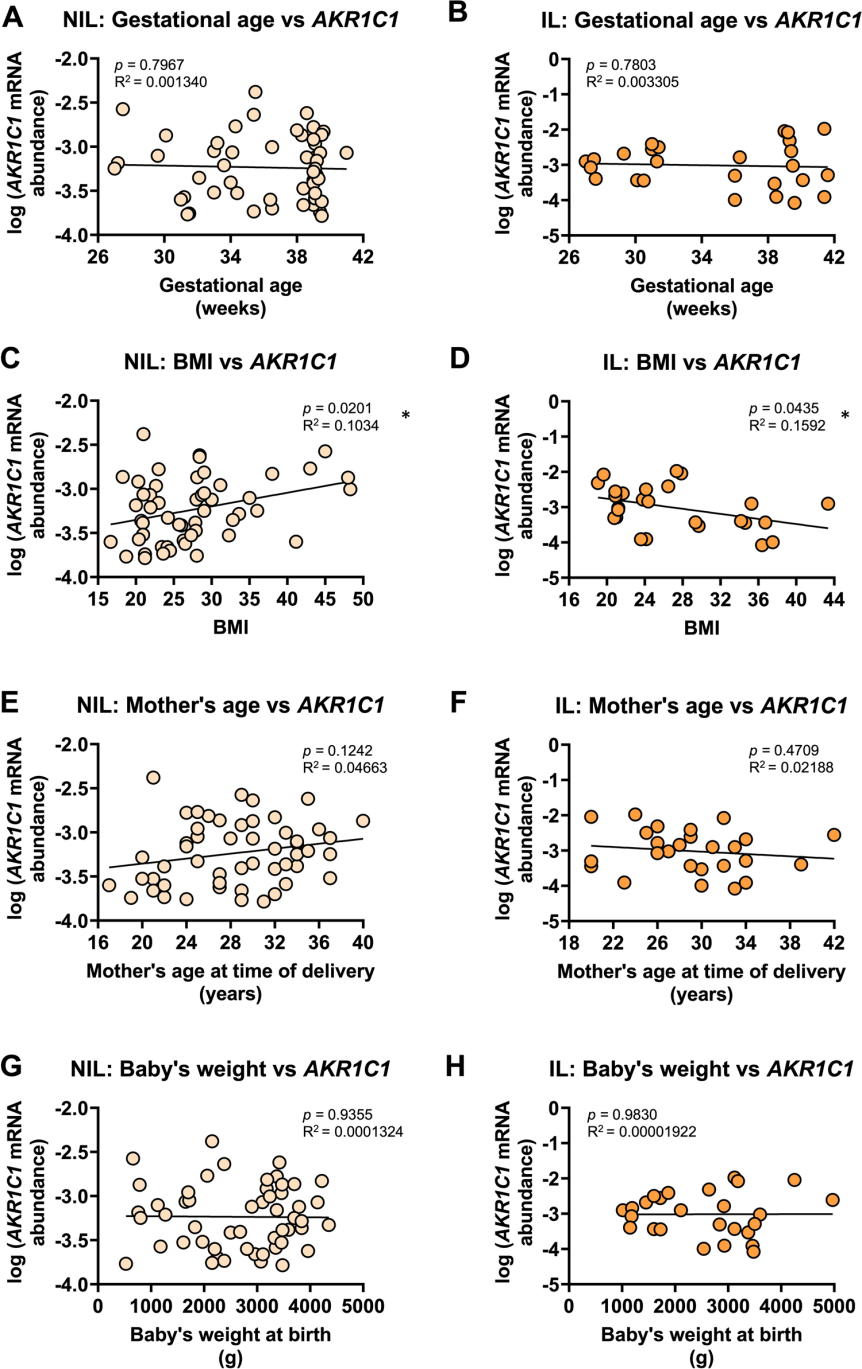
Analysis of pregnant human myometrial *AKR1C1* expression with respect to clinical data. Relative abundance of *AKR1C1* mRNA was measured in NIL (*n*=52) and IL myometrium (*n*=26) and expressed relative to Alien reference. **A** *AKR1C1* mRNA abundance across NIL gestation time points. **B** *AKR1C1* mRNA abundance across IL gestation time points. **C** *AKR1C1* mRNA abundance in NIL myometrium against BMI. **D** *AKR1C1* mRNA abundance in IL myometrium against BMI. **E** *AKR1C1* mRNA abundance in NIL myometrium against mother’s age at the time of delivery. **F** *AKR1C1* mRNA abundance in IL myometrium against mother’s age at the time of delivery. **G** *AKR1C1* mRNA abundance in NIL myometrium against baby’s weight at birth. **H** *AKR1C1* mRNA abundance in IL myometrium against baby’s weight at birth. *Data were log-transformed to reach normality and then analyzed by Pearson correlation tests*

Next, we examined *AKR1C1* expression with respect to other clinical characteristics. There was no significant relationship between *AKR1C1* mRNA abundance and gestational age at delivery (preterm and term combined) for NIL myometrium (*r*^2^=0.001, *p*=0.7967) (Fig. 3A) or IL myometrium (*r*^2^=0.003, *p*=0.7803) (Fig. 3B). We identified a statistically significant *positive* correlation between *AKR1C1* mRNA abundance and the BMI of the mother in NIL myometrium (*r*^2^=0.10, *p*=0.0201) (Fig. 3C), and a statistically significant *negative* correlation in IL myometrium (*r*^2^=0.16, *p*=0.0435) (Fig. 3D). No significant relationship was found between *AKR1C1* mRNA abundance and the mother’s age at the time of delivery for NIL myometrium (*r*^2^=0.05, *p*=0.1242) (Fig. 3E) or IL myometrium (*r*^2^=0.02, *p*=0.4709) (Fig. 3F). As expected, babies’ weights correlated with gestational age at delivery (*r*^2^=0.76, *p*<0.0001, data not shown), but there was no significant relationship between myometrial *AKR1C1* mRNA abundance and the baby’s birth weight, regardless of labor status (NIL, *r*^*2*^=0.0001, *p*=0.9355, Fig. 3G; IL. *r*^2^=0.00002, *p*=0.9830, Fig. 3H).

### *AKR1C1* mRNA Abundance and Sex of the Baby

**Fig. 4:**
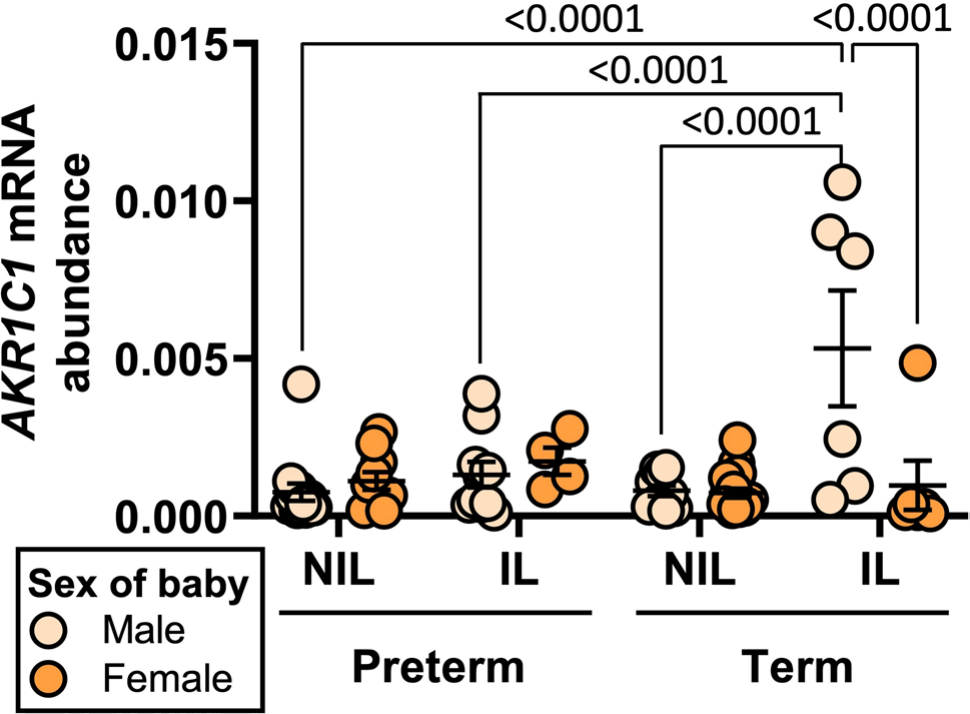
*AKR1C1* expression in myometrium from women who delivered male or female babies either preterm or at term. Relative abundance of *AKR1C1* mRNA was measured in the myometrium of women who delivered a male baby preterm NIL (*n*=14), a female baby preterm NIL (*n*=10), a male baby preterm IL (n=10), a female baby preterm IL (*n*=4), a male baby at term NIL (*n*=10), a female baby term NIL (*n*=18), a male baby term IL (*n*=6), or a female baby term IL (*n*=6). *AKR1C1* mRNA abundance is expressed relative to Alien reference. *Data were analyzed by two-way ANOVA with multiple comparisons (Sidak)*. Significant (*p*) values are indicated

We then analyzed myometrial *AKR1C1* mRNA abundance according to the sex of the baby. Among women who delivered male babies, *AKR1C1* mRNA abundance was significantly higher in term IL myometrium (*n*=6) compared to term NIL myometrium (*n*=10) (*p<*0.0001), preterm IL myometrium (*n*=10) (*p<*0.0001), and preterm NIL myometrium (*n*=14) (*p<*0.0001) (Fig. 4). Among women who delivered female babies, there were no statistically significant differences in myometrial *AKR1C1* mRNA abundance across labor status (NIL vs IL) and between preterm or term deliveries. Comparing across delivery of male and female babies, myometrial *AKR1C1* mRNA abundance was significantly higher in IL women who delivered males babies at term (*n=*6) compared to IL women who delivered female babies at term (*n*=6) (*p*<0.0001) (Fig. 4).

**Fig. 5:**
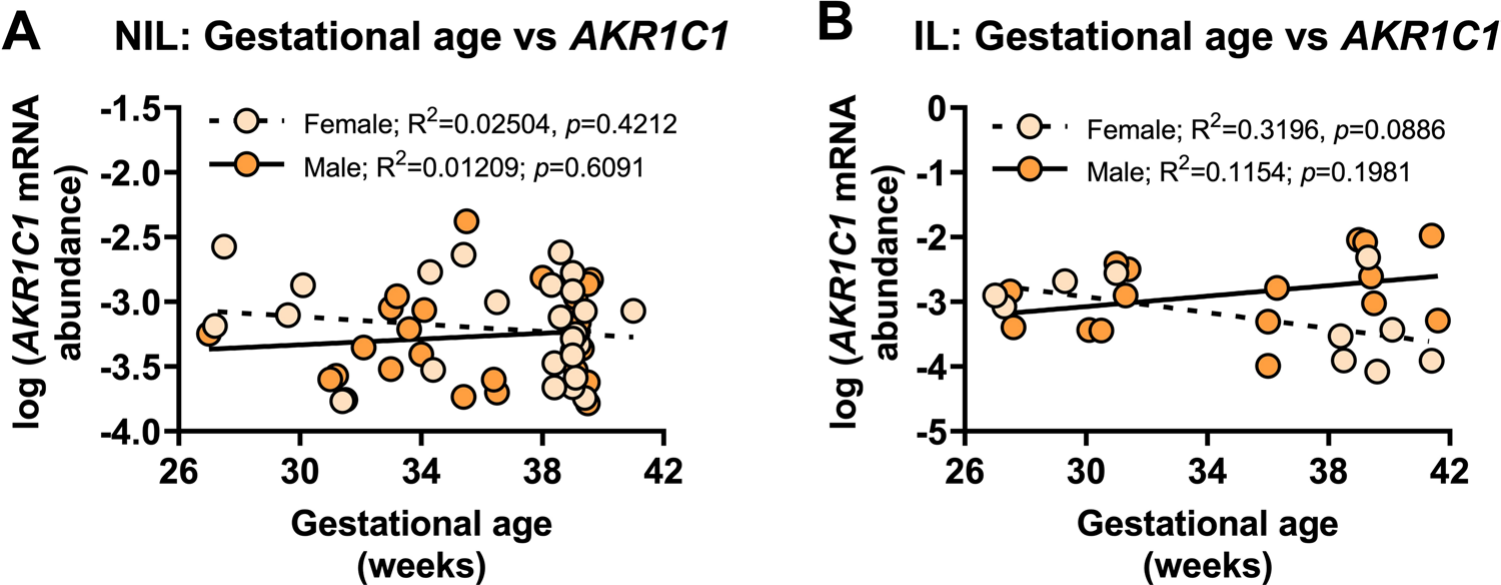
Analysis of *AKR1C1* expression in myometrium from women who delivered male or female babies with respect to gestational age (weeks). Relative abundance of *AKR1C1* mRNA was measured in NIL myometrium from women who delivered male (*n*=24) or female babies (*n*=28) and IL myometrium from women who delivered male (*n*=16) or female babies (*n*=10) and expressed relative to Alien reference. **A** *AKR1C1* mRNA abundance across NIL gestation time points. **B** *AKR1C1* mRNA abundance across IL gestation time points. *Data were log-transformed to reach normality and then analyzed by Pearson correlation tests*

There was no significant relationship between *AKR1C1* mRNA abundance and gestational age for NIL myometrium (preterm and term combined) from women who delivered male (*r*^2^=0.01, *p*=0.6091) or female babies (*r*^2^=0.03, *p*=0.4212) (Fig. 5A). Similarly, no significant relationship was found between *AKR1C1* mRNA abundance and gestational age in IL (preterm and term combined) myometrium from women who delivered male (*r*^2^=0.12, *p*=0.1981) or female babies (*r*^2^=0.32, *p*=0.0886) (Fig. 5B).

**Fig. 6:**
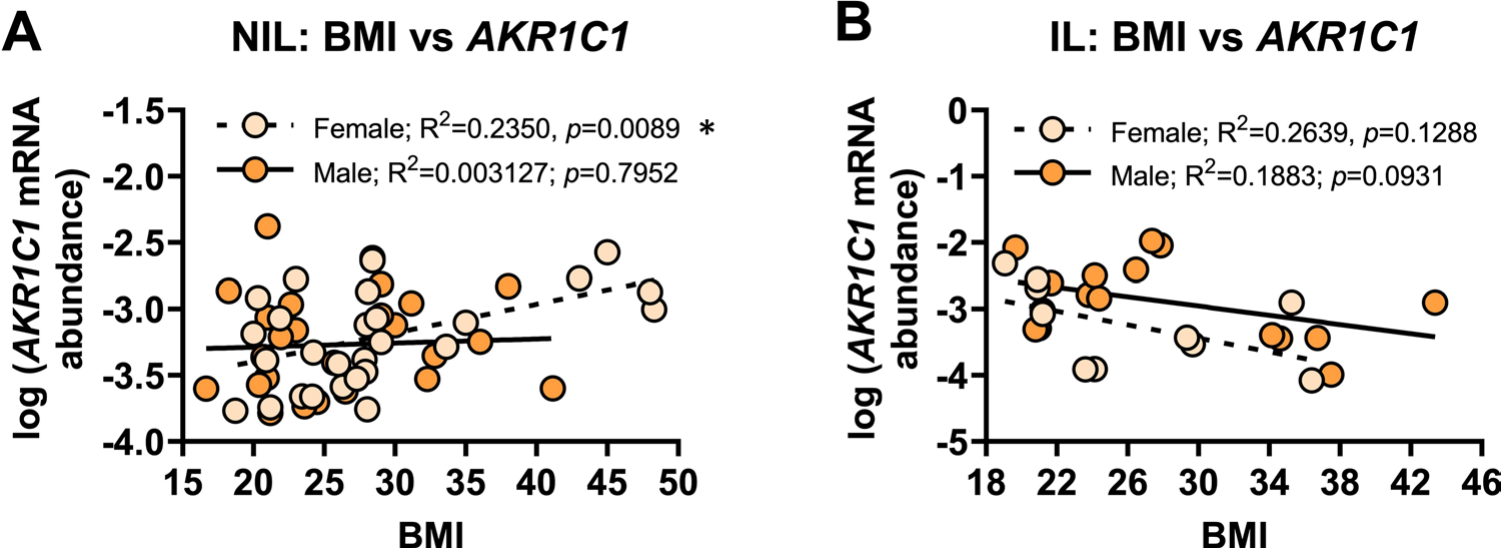
Analysis of *AKR1C1* expression in myometrium from women who delivered male or female babies with respect to mother’s BMI. Relative abundance of *AKR1C1* mRNA was measured in NIL myometrium from women who delivered male (*n*=24) or female babies (*n*=28) and IL myometrium from women who delivered male (*n*=16) or female babies (*n*=10) and expressed relative to Alien reference. **A** *AKR1C1* mRNA abundance in NIL myometrium against BMI. **B** *AKR1C1* mRNA abundance in IL myometrium against BMI. *Data were log-transformed to reach normality and then analyzed by Pearson correlation tests*

Upon examining patient BMI, recorded during second trimester, no significant relationship was found between *AKR1C1* mRNA abundance and BMI for NIL myometrium from women who delivered male babies (*r*^2^=0.003, *p*=0.7952); however, there was a statistically significant *positive* correlation between *AKR1C1* mRNA abundance and the BMI in NIL myometrium from women who delivered female babies (*r*^2^=0.235, *p*=0.0089) (Fig. 6A). No significant relationship was found between *AKR1C1* mRNA abundance and BMI in IL myometrium from women who delivered male (*r*^2^=0.19, *p*=0.0931) or female babies (*r*^2^=0.26, *p*=0.1288) (Fig. 6B).

**Fig. 7:**
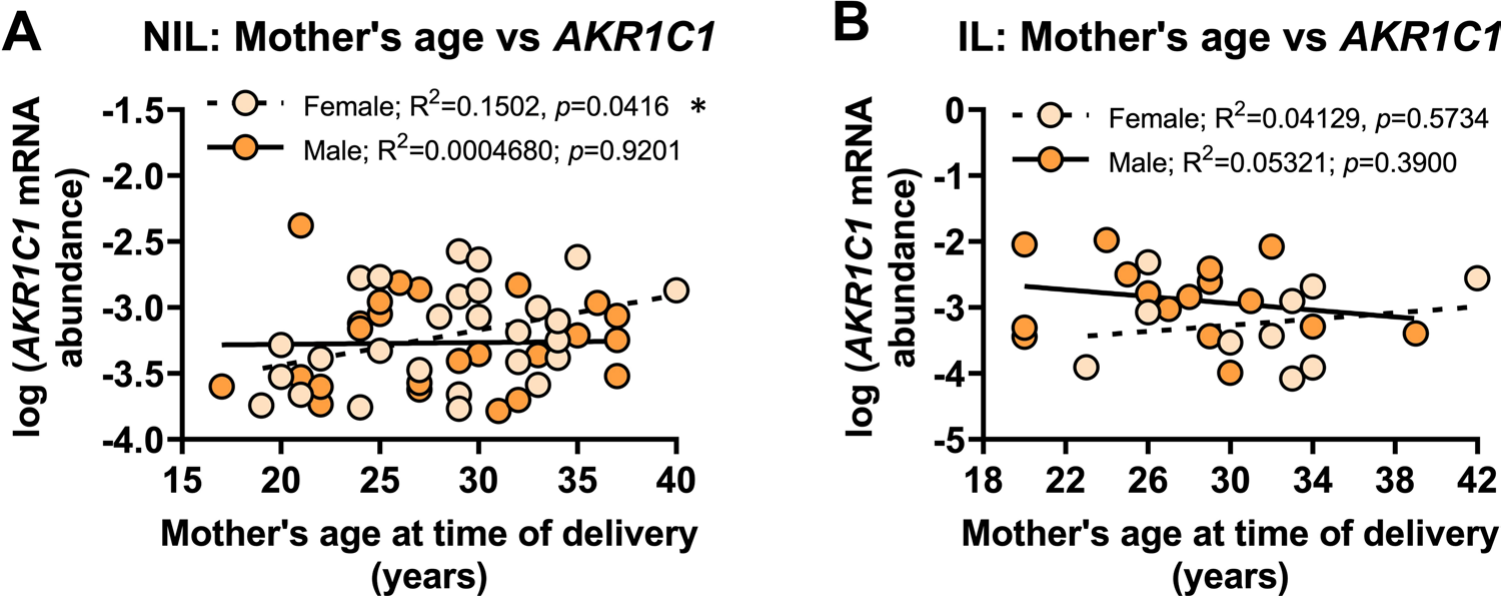
Analysis of *AKR1C1* expression in myometrium from women who delivered male or female babies with respect to mother’s age (years). Relative abundance of *AKR1C1* mRNA was measured in NIL myometrium from women who delivered male (*n*=24) or female babies (*n*=28) and IL myometrium from women who delivered male (*n*=16) or female babies (*n*=10) and expressed relative to Alien reference. **A** *AKR1C1* mRNA abundance in NIL myometrium against mother’s age at the time of delivery. **B** *AKR1C1* mRNA abundance in IL myometrium against mother’s age at the time of delivery. *Data were log-transformed to reach normality and then analyzed by Pearson correlation tests*

For mother’s age, no significant relationship was found between *AKR1C1* mRNA abundance and the mother’s age in NIL myometrium from women who delivered male babies (*r*^2^=0.0004, *p*=0.9201) (Fig. 7A); however, a statistically significant *positive* correlation was found between *AKR1C1* mRNA abundance and the mother’s age in NIL myometrium from women who delivered female babies (*r*^2^=0.15, *p*=0.0416) (Fig. 7A). Mother’s age did not significantly correlate with *AKR1C1* mRNA abundance in myometrium from IL deliveries of either male (*r*^2^=0.05, *p*=0.3900) or female babies (*r*^2^=0.04, *p*=0.5734) (Fig. 7B).

**Fig. 8:**
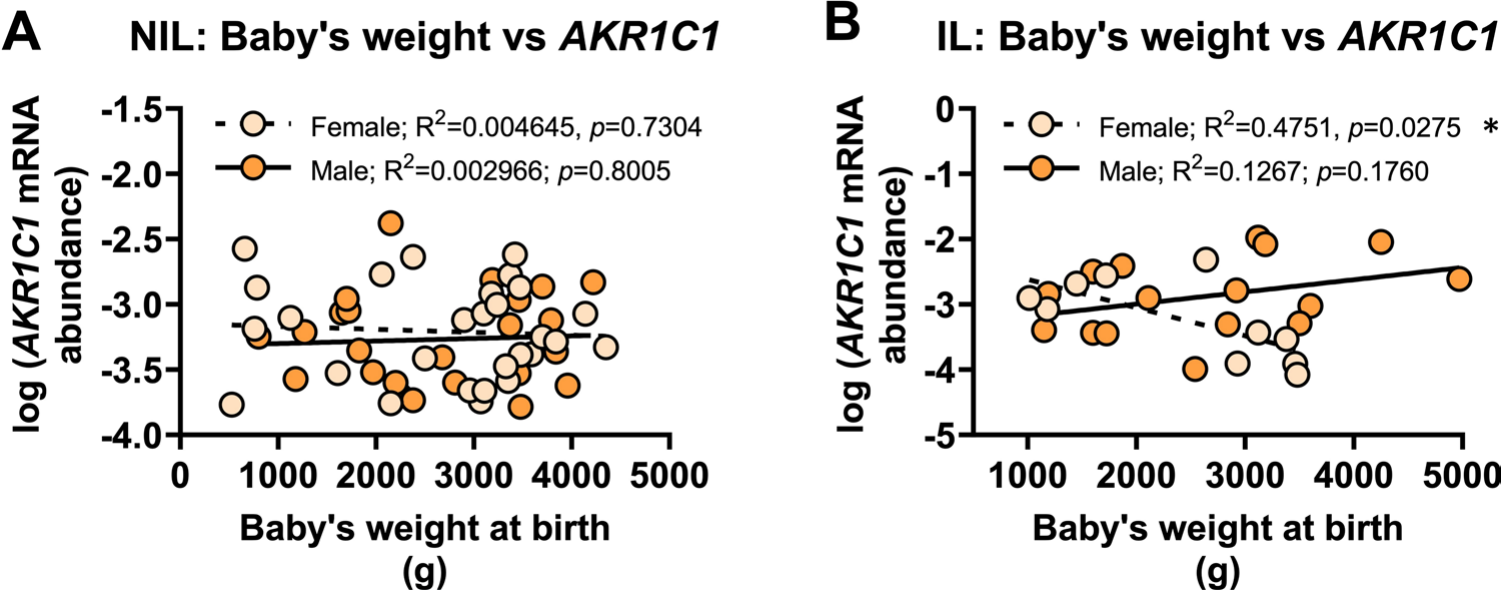
Analysis of *AKR1C1* expression in myometrium from women who delivered male or female babies with respect to baby’s weight at birth. Relative abundance of *AKR1C1* mRNA was measured in NIL myometrium from women who delivered male (*n*=24) or female babies (*n*=28) and IL myometrium from women who delivered male (*n*=16) or female babies (*n*=10) and expressed relative to Alien reference. **A** *AKR1C1* mRNA abundance in NIL myometrium against baby’s weight at birth. **B** *AKR1C1* mRNA abundance in IL myometrium against baby’s weight at birth. *Data were log-transformed to reach normality and then analyzed by Pearson correlation tests*

Predictably, there was no significant relationship between *AKR1C1* mRNA abundance and the baby’s birth weight in NIL myometrium from women who delivered male (*r*^2^=0.0029, *p*=0.8005) or female babies (*r*^2^=0.0046, *p*=0.7304) (Fig. 8A). Similarly, no significant relationship was found between *AKR1C1* mRNA abundance and the baby’s birth weight in IL myometrium from women who delivered male babies (*r*^2^=0.051, *p*=0.3847); however, there was a statistically significant *negative* correlation between *AKR1C1* mRNA abundance and the baby’s birth weight in IL myometrium from women who delivered female babies (*r*^2^=0.48, *p*=0.0275) (Fig. 8B).

### *AKR1C1* mRNA Abundance and Common Pregnancy Complications

Upon examining the presence of common pregnancy complications, we found that *AKR1C1* mRNA abundance was not affected by the presence or absence of IUGR (*n*=15) or GDM (*n*=3) in preterm NIL samples (data not shown).

### AKR1C1 Protein Levels

We ran a concentration gradient of S9 fraction from human liver (0, 0.25, 0.5, 1.0, 2.0, 3.0, 4.0, 5.0 μg/lane), as AKR1C1 is highly expressed in human liver and the S9 fraction is “a rich source of drug metabolizing enzymes” from human hepatocytes. When the concentration gradient blots were probed with either GeneTex anti-AKR1C1 or ThermoFisher anti-AKR1C1, each antibody revealed a single immunoreactive band of the expected molecular weight (37 kDa) for human AKR1C1 (see supplementary Figures S1 and S2), suggesting that S9 fraction from human liver was appropriate for use as a positive control. As a negative control, our gels/blots incorporated SDS protein extract from term, human placenta, which does not express AKR1C1 [24]. This was confirmed via probing a concentration gradient blot of human placenta protein extract (0.5 - 50 μg protein/lane), which revealed that an immunoreactive band of the expected molecular weight for AKR1C1 (~37 kDa) was not detected, even at high protein levels (i.e. 50 μg protein/lane) (see Supplementary Figure S3).

Western blotting of pregnant human myometrial protein extracts revealed that neither the GeneTex nor ThermoFisher anti-AKR1C1 antibodies were specific for AKR1C1, in that both antibodies cross-reacted with a triplicate of protein bands in the vicinity of the expected molecular weight of AKR1C1 (37 kDa) (see Supplementary Figures S4 and S5). Due to the lack of a reliable antibody, coupled with low AKR1C1 protein abundance in pregnant human myometrium necessitating the overloading of protein gels (50 μg protein/lane), gene expression data were unable to be confirmed at the protein level.

## Discussion

Prior studies have shown that *AKR1C1* mRNA abundance and 20α-HSD protein levels increase with labor onset in human myometrium at term [16, 20]. Our data are consistent with these findings as we have detected an average 6-fold increase in myometrial *AKR1C1* mRNA abundance in association with labor onset at term (Fig. 1). Our study is the first to examine myometrial *AKR1C1* expression during preterm birth and the relationship of *AKR1C1* expression with clinical characteristics of pregnant women.

Chorioamnionitis is one of the most common antecedents of preterm birth [1] and is present in approximately 66% of preterm births at 24 weeks or earlier, with this figure decreasing to 16% by 34 weeks [25]. Chorioamnionitis was present among 75% of our extreme preterm birth patients (<28 weeks) and 43% of our very preterm birth patients (28–32 weeks). Remarkably, *AKR1C1* mRNA abundance was significantly higher in the myometrium of preterm IL women who had clinical signs of chorioamnionitis, compared to the myometrium of preterm IL women with no evidence of chorioamnionitis. Inflammation has been robustly established as a driver of pro-labor gene expression [6, 26, 27] and our findings suggest that *AKR1C1* is upregulated in association with inflammatory signaling during premature labor. This is consistent with the findings of Nadeem et al. [28], who showed that pro-inflammatory mediators (lipopolysaccharide, 12-O-tetradecanoylphorbol-13-acetate) upregulated *AKR1C1* expression and increased 20α-HSD protein levels in immortalized human myometrial cells. Moreover, Nadeem et al. [28] demonstrated that *AKR1C1* expression was driven by the transcription factors, nuclear factor kappa-light-chain-enhancer of activated B cells (NF-кB) and activator protein 1 (AP-1). Combined with our data, these findings suggest that NF-кB and AP-1 likely drive *AKR1C1* expression during chorioamnionitis-driven preterm birth.

Maternal overweight and obesity is associated with an increased risk of pregnancy complications, including miscarriage, gestational hypertension, preeclampsia, gestational diabetes mellitus, preterm birth, induction of labor, cesarean section, and anesthetic complications [29–33]. Babies born to overweight and obese women are at greater risk of being born large for gestational age, have a higher incidence of congenital defects, and are at greater risk of developing obesity and metabolic disorders in childhood [29–32]. We have previously reported that maternal obesity is associated with dysregulated expression and function of hERG potassium channels [34], which likely contributes to the weak contractions and poor labor outcomes observed for many obese women. In this study, our data indicate that women who are not yet in labor have low levels of myometrial *AKR1C1* expression (Fig. 1), but that *AKR1C1* expression increases as BMI increases (Fig. 3C). Interestingly, this correlation was only observed in NIL women that delivered female babies. The implications of this relationship are not yet clear; however, it is tempting to speculate that it may contribute toward the increased risk of preterm birth among overweight and obese women through increasing 20α-HSD levels, thus promoting intracellular P4 metabolism. In a nationwide cohort study of more than 1.5 million deliveries in Sweden from 1992 to 2010, Cnattingius et al. [33] identified that maternal obesity had a dose-response relationship specifically in relation to spontaneous extremely preterm delivery (preterm contractions and preterm premature rupture of membranes). Unfortunately, the study did not examine preterm birth rates on the basis of fetal sex, meaning the link between preterm birth and obesity was not specifically associated with the delivery of female babies, as per our linkage of obesity to myometrial *AKR1C1* expression.

Studies of both Western [35–38] and non-Western populations [39, 40] have found that women carrying male fetuses are at greater risk of preterm birth. In our study, we found no significant difference in myometrial *AKR1C1* expression between preterm deliveries of male versus female babies. We did, however, find significantly higher levels of myometrial *AKR1C1* expression in association with delivery of males at term versus females at term. Although these data do not directly link myometrial *AKR1C1* expression to the increased risk of preterm birth for pregnancies carrying male fetuses, the data suggests that myometrial P4 metabolism becomes more intense towards term in pregnancies carrying a male fetus compared to females, which may contribute to the increased risk of early labor. Our observations represent an initial step to identify a mechanism responsible for the male sex bias of preterm birth.

*AKR1C1* and its paralogs, potentially, are involved in the process of functional P4 withdrawal at term human parturition. Involvement of *AKR1C1* in pregnancy complications related to obesity, and potentially preterm birth, are indicated by our data and warrant further studies. Additionally, our data provide another instance of sex-related differences documented between women who delivered female versus male babies. Other instances include human chorionic gonadotropin (hCG) being significantly higher in women who delivered female babies [41] and more proinflammatory/pro-angiogenic immune milieu observed in women who delivered male babies [42]. Further studies are necessary to determine the exact reasons for the sex differences observed in this study and whether there are any links to other known sex-related differences.

A strength of this study is that we have analyzed tissue samples frozen immediately after delivery from well-phenotyped women. The results, therefore, closely reflect the *in vivo* state allowing insights into the physiologic and pathologic conditions influencing *AKR1C1* gene activity.

A limitation of the study is the lack of confirmation of gene expression data at the protein level. Extensive efforts were made to analyze AKR1C1 protein levels in extracts of pregnant human myometrium via Western blotting; however, this proved unfruitful due to both the assessed anti-AKR1C1 antibodies cross-reacting with a triplicate of unknown gene products within the vicinity of the expected molecular weight for human AKR1C1. It is possible that the triplicate of protein bands detected may correspond to AKR1C1 (37 kDa), AKR1C2 (36 kDa), and AKR1C3 (34 kDa), given that in humans, AKR1C1 shares 97.8% and 87.9% sequence homology with AKR1C2 and AKR1C3, respectively (see Supplementary Figure S6 for amino acid sequence alignments). Nonetheless, the precise identity of the triplicate gene products remains currently unknown. Therefore, it remains to be confirmed whether the reported AKR1C1 expression differences at the mRNA level are manifest at the protein level. Another limitation is the low number in certain patient groups, for instance, the preterm IL clinical chorioamnionitis group (*n*=6). Statistically significant differences have been detected despite the limited power, but further sample collection is still warranted to confirm our results. Furthermore, the classification of samples with chorioamnionitis is based on a clinical diagnosis of genital tract inflammation. Therefore, although unlikely, subclinical microbial-associated chorioamnionitis may still exist among samples classified as free of inflammation.

## Conclusions

In this study, we demonstrate for the first time the presence of *AKR1C1* expression in both preterm and term pregnant human myometrium before and during labor. The onset of labor at term was associated with upregulated myometrial *AKR1C1* expression and was observed only in women who delivered male babies. Initial results indicate an association between chorioamnionitis and myometrial *AKR1C1* expression. Overall, our results are the first to draw a link between *AKR1C1* expression in pregnant human myometrium, obesity and fetal gender. The new information will advance our understanding of how obesity leads to dysfunctional labor in women carrying a male or female fetus.

## Supplementary Information

ESM 1 **Figure S1.** Probing of S9 Fraction from human liver with GeneTex anti-AKR1C1. S9 fraction from human liver extract (0, 0.25, 0.5, 1.0, 2.0, 3.0, 4.0, 5.0 μg/lane) was separated by 1D SDS-PAGE then transferred to nitrocellulose membrane. Total protein was visualized by Ponceau S staining then imaged (left panel). Membranes were then probed for AKR1C1 detection (right panel) using GeneTex (cat# GTX105620) rabbit anti-AKR1C1 polyclonal antibody (1:1000) and anti-rabbit-HRP secondary antibody (1:2000). Representative image shows immunoreactive bands detected after 1 min exposure using Immobilon Forte chemiluminescence reagent. Molecular weight marker was Novex™ Sharp Pre-stained Protein Standard.

ESM 2 **Figure S2.** Probing of S9 Fraction from human liver with ThermoFisher anti-AKR1C1. S9 fraction from human liver extract (0, 0.25, 0.5, 1.0, 2.0, 3.0, 4.0, 5.0, 10, 20 μg/lane) was separated by 1D SDS-PAGE then transferred to nitrocellulose membrane. Total protein was visualized by Ponceau S staining then imaged (left panel). Membranes were then probed for AKR1C1 detection (right panel) using ThermoFisher (cat# PA5-84776) rabbit anti-AKR1C1 polyclonal antibody (1:1000) and anti-rabbit-HRP secondary antibody (1:2000). Representative image shows immunoreactive bands detected after 1 min exposure using Immobilon Forte chemiluminescence reagent. Molecular weight marker was Novex™ Sharp Pre-stained Protein Standard.

ESM 3 **Figure S3.** Probing of human placenta protein extracts with GeneTex anti-AKR1C1. Protein extract from human liver (0, 0.5, 1.0, 2.0, 3.0, 4.0, 5.0, 10, 20, 50 μg/lane) was separated by 1D SDS-PAGE then transferred to nitrocellulose membrane. Total protein was visualized by Ponceau S staining then imaged (left panel). Membranes were then probed for AKR1C1 detection (right panel) using GeneTex (cat# GTX105620) rabbit anti-AKR1C1 polyclonal antibody (1:1000) and anti-rabbit-HRP secondary antibody (1:2000). Representative image shows immunoreactive bands detected after 3 min exposure using Immobilon Forte chemiluminescence reagent. Molecular weight marker was Novex™ Sharp Pre-stained Protein Standard.

ESM 4 **Figure S4.** Probing of myometrial protein extracts with GeneTex anti-AKR1C1. Protein extracts from term NIL myometrium (*n*=9) were separated by SDS-PAGE then transferred to nitrocellulose membrane (50 μg/lane). S9 Fraction from human liver extract (0.5 μg/lane) and human placenta extract (50 μg/lane) were included as positive and negative controls, respectively. Total protein was visualized by Ponceau S staining then imaged (left panel). Membranes were then probed for AKR1C1 detection (right panel) using GeneTex (cat# GTX105620) rabbit anti-AKR1C1 polyclonal antibody (1:1000) and anti-rabbit-HRP secondary antibody (1:2000). Representative image shows immunoreactive bands detected after 3 min exposure using Immobilon Forte chemiluminescence reagent. Blots were then stripped and re-probed using mouse anti-β-actin (1:1000) and anti-mouse-HRP (1:2000) (15 sec exposure). Molecular weight marker was Novex™ Sharp Pre-stained Protein Standard.

ESM 5 **Figure S5.** Probing of myometrial protein extracts with ThermoFisher anti-AKR1C1. Protein extracts from term NIL (*n*=2), preterm NIL (*n*=2), term IL (*n*=2), and preterm IL (*n*=2) myometrium were separated by SDS-PAGE then transferred to nitrocellulose membrane (50 μg/lane). S9 Fraction from human liver extract (0.5 μg/lane) was included as a positive control, while extracts from human placenta (50 μg/lane) and mouse testis (50 μg/lane) were included as negative controls. Total protein was visualized by Ponceau S staining then imaged (left panel). Membranes were then probed for AKR1C1 detection (right panel) using ThermoFisher (cat# PA5-84776) rabbit anti-AKR1C1 polyclonal antibody (1:1000) and anti-rabbit-HRP secondary antibody (1:2000). Representative image shows immunoreactive bands detected after 11 min exposure using Immobilon Forte chemiluminescence reagent. Blots were then stripped and re-probed using mouse anti-β-actin (1:1000) and anti-mouse-HRP (1:2000) (15 sec exposure). Molecular weight marker was Novex™ Sharp Pre-stained Protein Standard.

ESM 6 **Figure S6.** Alignment of AKR1C1 amino acid sequence against AKR1C2 and AKR1C3. Alignment of the amino acid sequence of AKR1C1 against AKR1C2 (upper) and AKR1C3 (lower) reveals that there only two differences in the amino acid sequence that distinguish AKR1C1 from both AKR1C2 and AKR1C3. Each of these differences (highlighted by blue boxes) are only a single amino acid substitution: a cysteine (AKR1C1) to serine (AKR1C2, AKR1C3) substitution at residue 87, and a valine (AKR1C1) to methionine substitution (AKR1C2, AKR1C3) at residue 151. GeneTex indicate that their anti-AKR1C1 antibody (cat# GTX105620) was raised against a *recombinant protein encompassing a sequence within the center region of human AKR1C1*. Therefore, GTX105620 could only be specific for AKR1C1 if its binding was contingent upon detecting the single amino acid substitution at residue 151 (at the center region of AKR1C1), which is highly unlikely for a polyclonal. ThermoFisher indicate that their anti-AKR1C1 antibody (cat# PA5-84776) was raised against an immunogen spanning amino acids 225 – 249 of human AKR1C1 (indicated by the red box). However, sequence alignment reveals no differences in the amino acid sequence between AKR1C1 and AKR1C2 within amino acids 225 – 249.

*NIL* not-in-labor, *IL* in-labor, *BMI* body mass index, *IUGR* intrauterine growth restriction, *GDM* gestational diabetes mellitus

*AKR1C1* Aldo-keto reductase family 1 member C1

## Data Availability

Data available on request due to privacy/ethical restrictions.

## Glossary

1D: one-dimensional
20α-HSD: 20α-hydroxysteroid dehydrogenase
20α-OHP: 20α-hydroxyprogesterone
AKR: aldo-keto reductase
*AKR1C1*: aldo-keto reductase family 1 member C1 gene
*AKR1C2*: aldo-keto reductase family 1 member C2 gene
*AKR1C3*: aldo-keto reductase family 1 member C3 gene
ANOVA: analysis of variance
AP-1: activator protein 1
BCA: bicinchoninic acid
BMI: body mass index
CAP: contraction-associated protein
GDM: gestational diabetes mellitus
Cx43: connexin 43
EDTA: ethylenediaminetetraacetic acid
*GJA1*: gap junction alpha-1 protein
HRP: horseradish peroxidase
IL: in-labor
IUGR: intrauterine growth restriction
miR-200: microRNA-200
mRNA: messenger ribonucleic acid
NF-кB: nuclear factor kappa-light-chain-enhancer of activated B cells
NIL: not-in-labor
P4: progesterone
PBS: phosphate-buffered solution
PR: progesterone receptor
RT-PCR: real-time quantitative polymerase chain reaction
RNA: ribonucleic acid
SDS: sodium dodecyl sulfate
SDS-PAGE: sodium dodecyl sulfate-polyacrylamide gel electrophoresis
STAT5b: signal transducer and activator of transcription 5b
TBS: tris buffered saline
TBS-T: tris buffered saline tween-20
