# Supplementary figures and images for: 20α-Hydroxysteroid Dehydrogenase Expression in the Human Myometrium at Term and Preterm Birth: Relationships to Fetal Sex and Maternal Body Mass Index
Source: Reprod Sci. 2023 Feb 10;30(8):2512–23. doi: 10.1007/s43032-023-01183-2 (PMC10354170; doi:10.1007/s43032-023-01183-2)

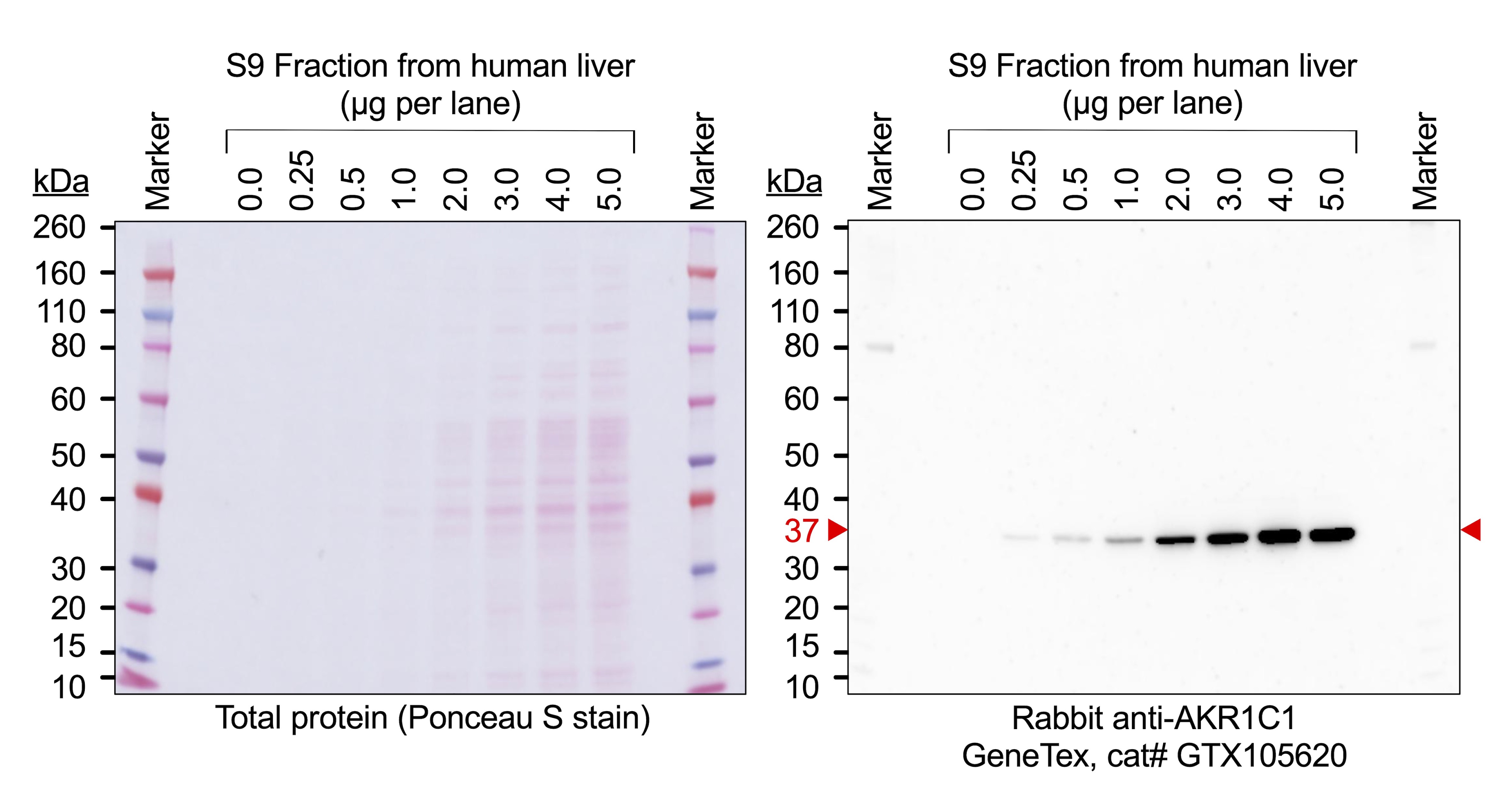

Supplement: Supplementary file 1 — Figure S1. Probing of S9 Fraction from human liver with GeneTex anti-AKR1C1. S9 fraction from human liver extract (0, 0.25, 0.5, 1.0, 2.0, 3.0, 4.0, 5.0 μg/lane) was separated by 1D SDS-PAGE then transferred to nitrocellulose membrane. Total protein was visualized by Ponceau S staining then imaged (left panel). Membranes were then probed for AKR1C1 detection (right panel) using GeneTex (cat# GTX105620) rabbit anti-AKR1C1 polyclonal antibody (1:1000) and anti-rabbit-HRP secondary antibody (1:2000). Representative image shows immunoreactive bands detected after 1 min exposure using Immobilon Forte chemiluminescence reagent. Molecular weight marker was Novex™ Sharp Pre-stained Protein Standard. [file 43032_2023_1183_MOESM1_ESM.jpg]

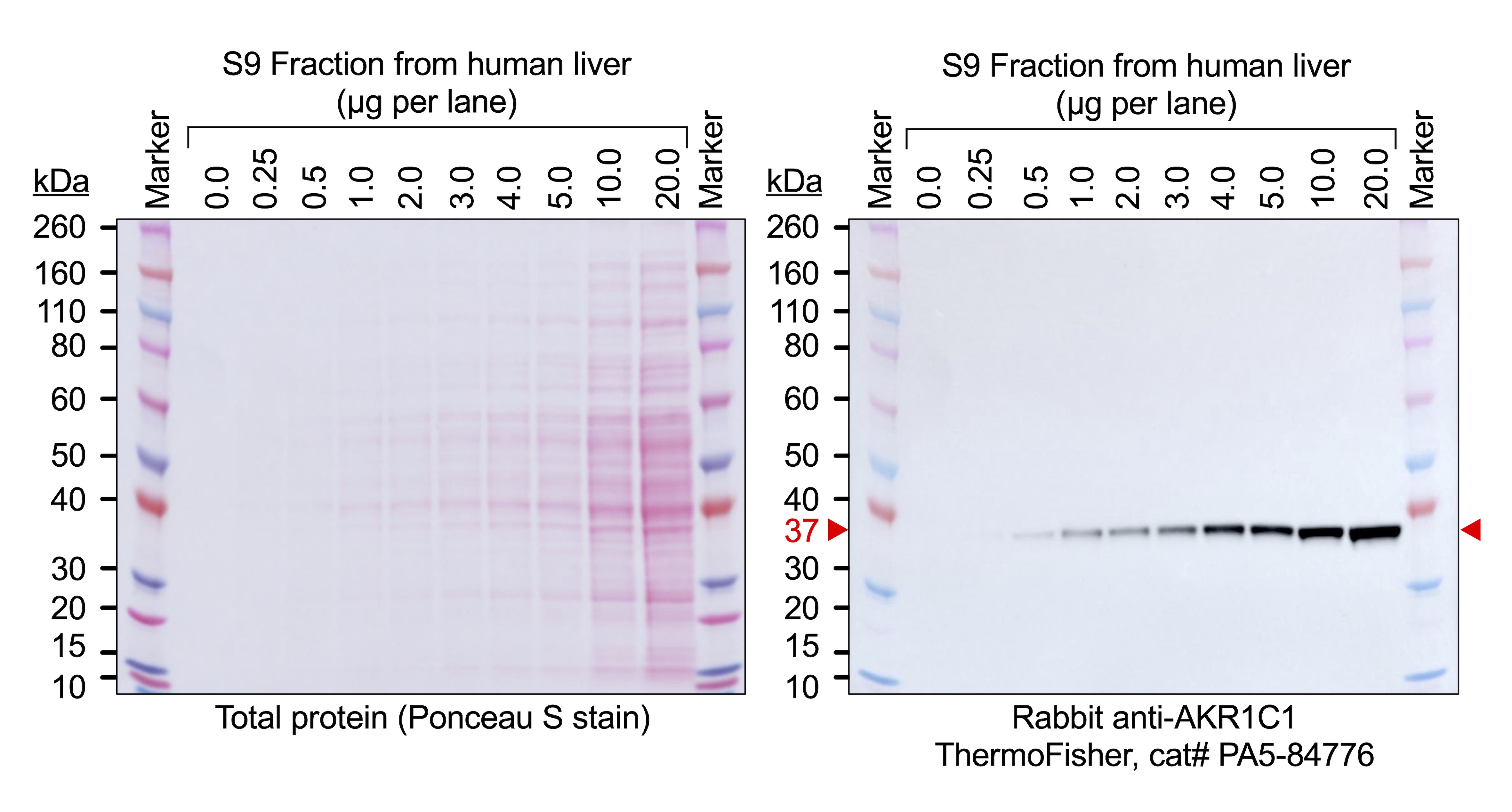

Supplement: Supplementary file 2 — Figure S2. Probing of S9 Fraction from human liver with ThermoFisher anti-AKR1C1. S9 fraction from human liver extract (0, 0.25, 0.5, 1.0, 2.0, 3.0, 4.0, 5.0, 10, 20 μg/lane) was separated by 1D SDS-PAGE then transferred to nitrocellulose membrane. Total protein was visualized by Ponceau S staining then imaged (left panel). Membranes were then probed for AKR1C1 detection (right panel) using ThermoFisher (cat# PA5-84776) rabbit anti-AKR1C1 polyclonal antibody (1:1000) and anti-rabbit-HRP secondary antibody (1:2000). Representative image shows immunoreactive bands detected after 1 min exposure using Immobilon Forte chemiluminescence reagent. Molecular weight marker was Novex™ Sharp Pre-stained Protein Standard. [file 43032_2023_1183_MOESM2_ESM.jpg]

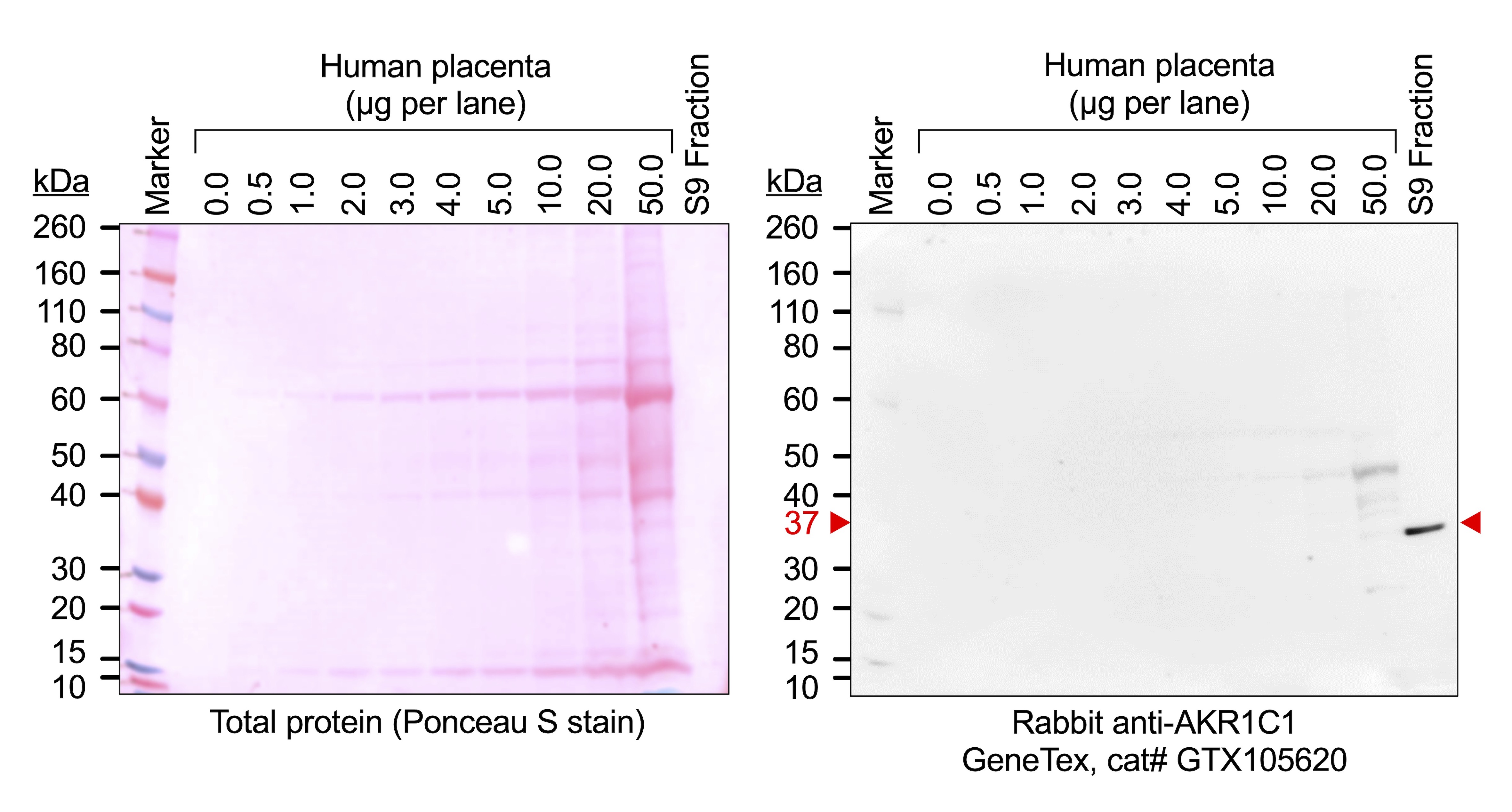

Supplement: Supplementary file 3 — Figure S3. Probing of human placenta protein extracts with GeneTex anti-AKR1C1. Protein extract from human liver (0, 0.5, 1.0, 2.0, 3.0, 4.0, 5.0, 10, 20, 50 μg/lane) was separated by 1D SDS-PAGE then transferred to nitrocellulose membrane. Total protein was visualized by Ponceau S staining then imaged (left panel). Membranes were then probed for AKR1C1 detection (right panel) using GeneTex (cat# GTX105620) rabbit anti-AKR1C1 polyclonal antibody (1:1000) and anti-rabbit-HRP secondary antibody (1:2000). Representative image shows immunoreactive bands detected after 3 min exposure using Immobilon Forte chemiluminescence reagent. Molecular weight marker was Novex™ Sharp Pre-stained Protein Standard. [file 43032_2023_1183_MOESM3_ESM.jpg]

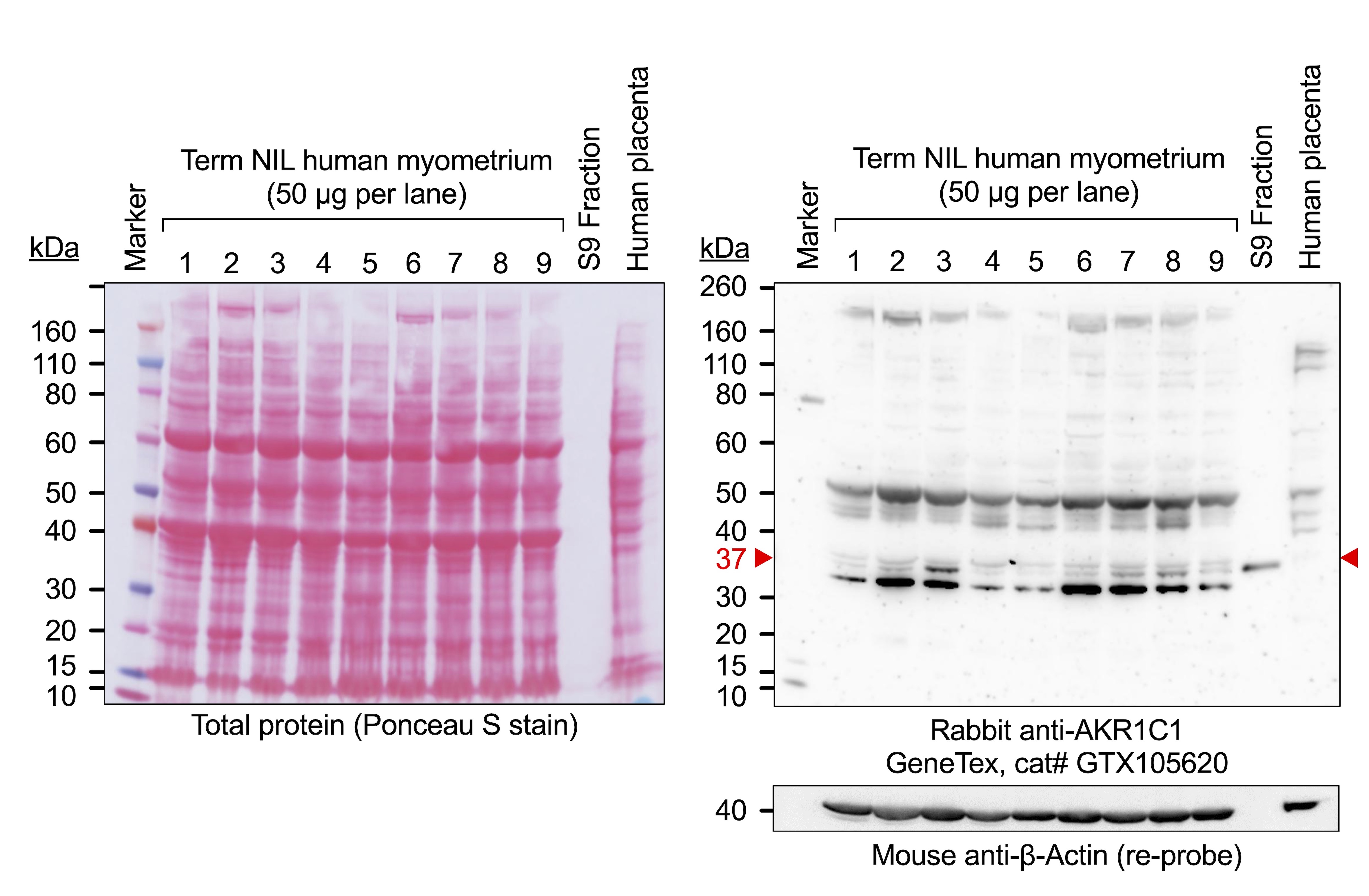

Supplement: Supplementary file 4 — Figure S4. Probing of myometrial protein extracts with GeneTex anti-AKR1C1. Protein extracts from term NIL myometrium (n=9) were separated by SDS-PAGE then transferred to nitrocellulose membrane (50 μg/lane). S9 Fraction from human liver extract (0.5 μg/lane) and human placenta extract (50 μg/lane) were included as positive and negative controls, respectively. Total protein was visualized by Ponceau S staining then imaged (left panel). Membranes were then probed for AKR1C1 detection (right panel) using GeneTex (cat# GTX105620) rabbit anti-AKR1C1 polyclonal antibody (1:1000) and anti-rabbit-HRP secondary antibody (1:2000). Representative image shows immunoreactive bands detected after 3 min exposure using Immobilon Forte chemiluminescence reagent. Blots were then stripped and re-probed using mouse anti-β-actin (1:1000) and anti-mouse-HRP (1:2000) (15 sec exposure). Molecular weight marker was Novex™ Sharp Pre-stained Protein Standard. [file 43032_2023_1183_MOESM4_ESM.jpg]

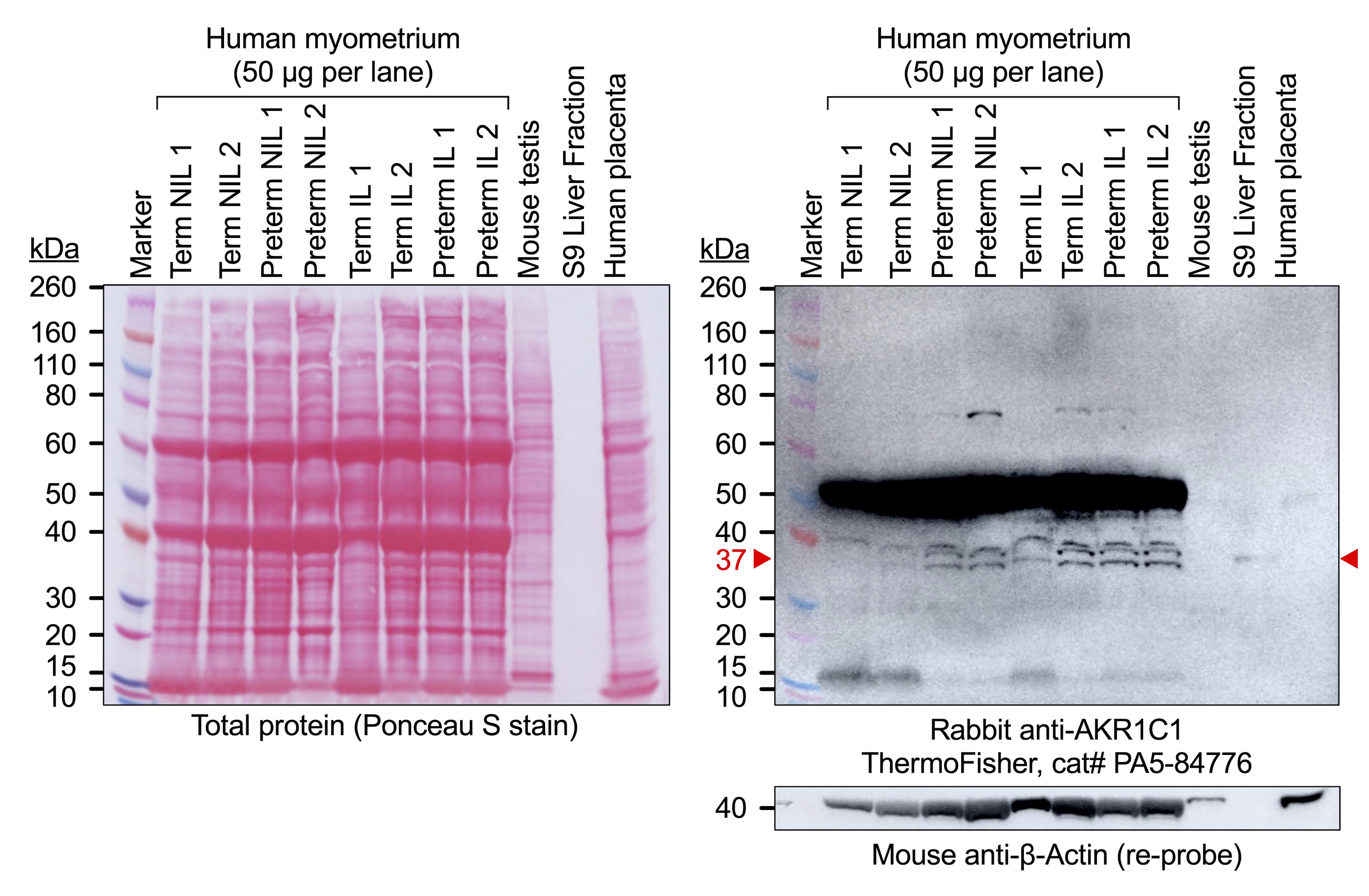

Supplement: Supplementary file 5 — Figure S5. Probing of myometrial protein extracts with ThermoFisher anti-AKR1C1. Protein extracts from term NIL (n=2), preterm NIL (n=2), term IL (n=2), and preterm IL (n=2) myometrium were separated by SDS-PAGE then transferred to nitrocellulose membrane (50 μg/lane). S9 Fraction from human liver extract (0.5 μg/lane) was included as a positive control, while extracts from human placenta (50 μg/lane) and mouse testis (50 μg/lane) were included as negative controls. Total protein was visualized by Ponceau S staining then imaged (left panel). Membranes were then probed for AKR1C1 detection (right panel) using ThermoFisher (cat# PA5-84776) rabbit anti-AKR1C1 polyclonal antibody (1:1000) and anti-rabbit-HRP secondary antibody (1:2000). Representative image shows immunoreactive bands detected after 11 min exposure using Immobilon Forte chemiluminescence reagent. Blots were then stripped and re-probed using mouse anti-β-actin (1:1000) and anti-mouse-HRP (1:2000) (15 sec exposure). Molecular weight marker was Novex™ Sharp Pre-stained Protein Standard. [file 43032_2023_1183_MOESM5_ESM.jpg]

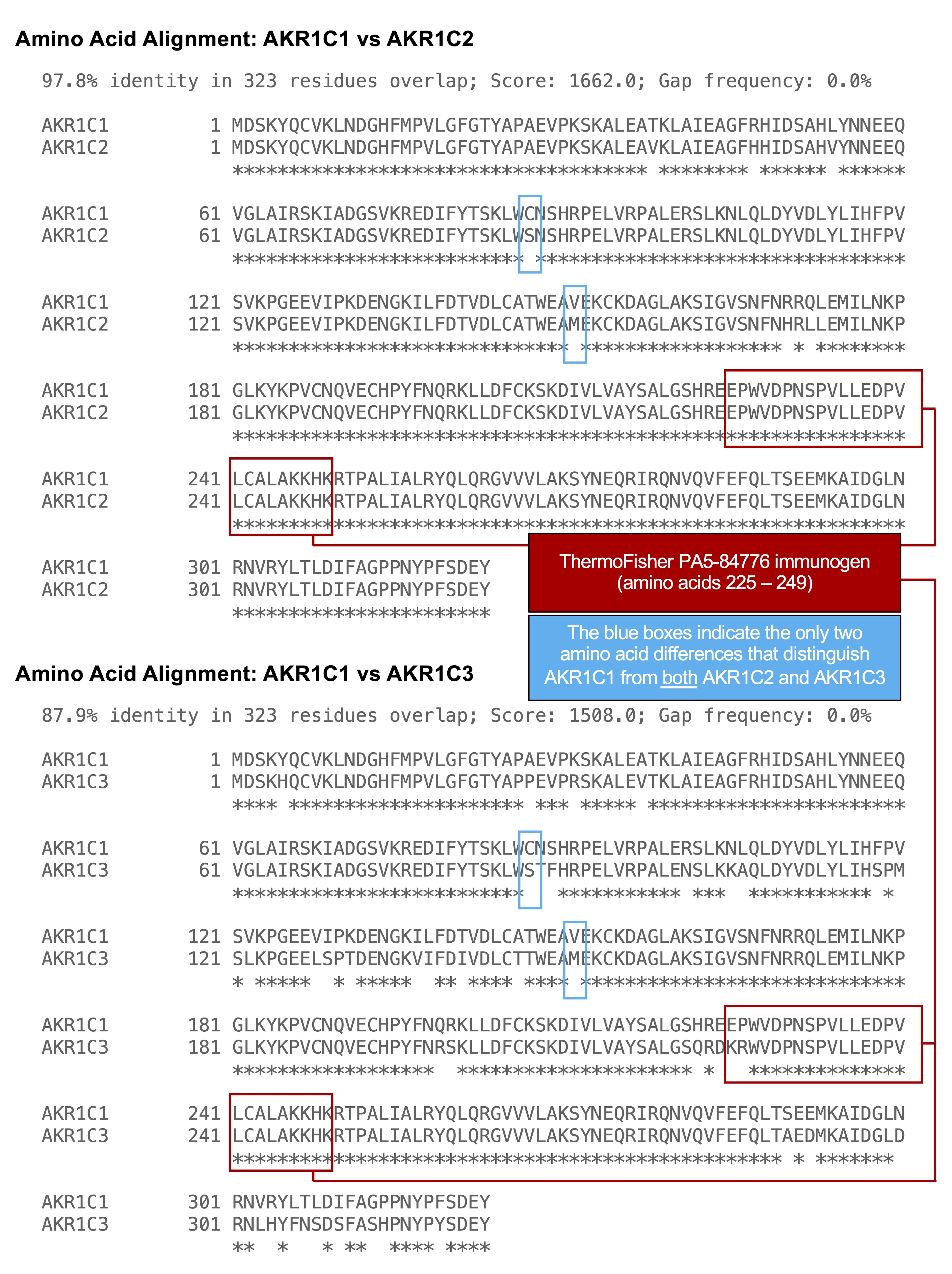

Supplement: Supplementary file 6 — Figure S6. Alignment of AKR1C1 amino acid sequence against AKR1C2 and AKR1C3. Alignment of the amino acid sequence of AKR1C1 against AKR1C2 (upper) and AKR1C3 (lower) reveals that there only two differences in the amino acid sequence that distinguish AKR1C1 from both AKR1C2 and AKR1C3. Each of these differences (highlighted by blue boxes) are only a single amino acid substitution: a cysteine (AKR1C1) to serine (AKR1C2, AKR1C3) substitution at residue 87, and a valine (AKR1C1) to methionine substitution (AKR1C2, AKR1C3) at residue 151. GeneTex indicate that their anti-AKR1C1 antibody (cat# GTX105620) was raised against a recombinant protein encompassing a sequence within the center region of human AKR1C1. Therefore, GTX105620 could only be specific for AKR1C1 if its binding was contingent upon detecting the single amino acid substitution at residue 151 (at the center region of AKR1C1), which is highly unlikely for a polyclonal. ThermoFisher indicate that their anti-AKR1C1 antibody (cat# PA5-84776) was raised against an immunogen spanning amino acids 225 – 249 of human AKR1C1 (indicated by the red box). However, sequence alignment reveals no differences in the amino acid sequence between AKR1C1 and AKR1C2 within amino acids 225 – 249. [file 43032_2023_1183_MOESM6_ESM.jpg]
